# Observational study of medical marijuana as a treatment for treatment‐resistant epilepsies

**DOI:** 10.1002/acn3.51537

**Published:** 2022-03-10

**Authors:** Orrin Devinsky, Angelica Marmanillo, Theresa Hamlin, Philip Wilken, Daniel Ryan, Conor Anderson, Daniel Friedman, George Todd

**Affiliations:** ^1^ NYU Comprehensive Epilepsy Center New York New York USA; ^2^ The Center for Discovery Harris New York USA; ^3^ Crystal Run Healthcare Rock Hill New York USA; ^4^ Icahn School of Medicine at Mount Sinai New York New York USA

## Abstract

**Objectives:**

Medical cannabis formulations with cannabidiol (CBD) and delta‐9‐tetrahydrocannabinol (THC) are widely used to treat epilepsy. We studied the safety and efficacy of two formulations.

**Methods:**

We prospectively observed 29 subjects (12 to 46 years old) with treatment‐resistant epilepsies (11 Lennox–Gastaut syndrome; 15 with focal or multifocal epilepsy; three generalized epilepsy) were treated with medical cannabis (1THC:20CBD and/or 1THC:50CBD; maximum of 6 mg THC/day) for ≥24 weeks. The primary outcome was change in convulsive seizure frequency from the pre‐treatment baseline to the stable optimal dose phase.

**Results:**

There were no significant differences during treatment on stable maximal doses for convulsive seizure frequency, seizure duration, postictal duration, or use of rescue medications compared to baseline. No benefits were seen for behavioral disorders or sleep duration; there was a trend for more frequent bowel movements compared to baseline. Ten adverse events occurred in 6/29 patients, all were transient and most unrelated to study medication. No serious adverse events were related to study medication.

**Interpretation:**

Our prospective observational study of two high‐CBD/low‐THC formulations found no evidence of efficacy in reducing seizures, seizure duration, postictal duration, or rescue medication use. Behavioral disorders or sleep duration was unchanged. Study medication was generally well tolerated. The doses of CBD used were lower than prior studies. Randomized trials with larger cohorts are needed, but we found no evidence of efficacy for two CBD:THC products in treating epilepsy, sleep, or behavior in our population.

## Introduction

Treatment‐resistant epilepsy (TRE) affects 30% of epilepsy patients and can progressively impair neurological function and quality of life, and cause injury and death from sudden unexpected death in epilepsy (SUDEP), and cause other seizure‐related (e.g. drowning), and indirect (e.g. metabolic disorder from medication side effects) consequences. Among young adult TRE patients, epilepsy kills 3%–6% per decade.^1^, ^2^ Most TRE patients suffer ongoing seizures despite disabling side effects from multiple anti‐seizure medications (ASMs). There is a dire need for new therapies exploiting novel mechanisms of action.

Cannabis (marijuana) species contain more than 500 compounds, including the cannabinoids Δ‐9‐tetrahydrocannabinol (THC) and cannabidiol (CBD). THC is the main psychoactive compound and CBD is the main non‐psychoactive compound in cannabis. Other cannabinoids and non‐cannabinoid molecules in cannabis have diverse biologic activities.^3^ Artisanal cannabis strains with high‐CBD:THC ratios gained widespread media attention to treat children with TREs such as Dravet Syndrome (DS) and Lennox–Gastaut Syndrome (LGS).^4^, ^5^ Small unblinded trials used different combinations of CBD, THC, and other cannabinoids, terpenes and flavonoids that rarely documented the precise contents, purity, or consistency of the products. A good manufacturing process (GMP) quality high‐CBD/low‐THC (50:1) formulation had comparable safety and efficacy in an open‐label study to treat seizures in Dravet syndrome^6^ as a 99% CBD formulation with <0.3% THC in open‐label and randomized controlled trials.^2^, ^5^ Randomized, placebo‐controlled trials have established the safety and efficacy of a 99% CBD formulation (Epidiolex®) to treat convulsive seizures in Dravet Syndrome, drop seizures in Lennox–Gastaut, and seizures in Tuberous Sclerosis Complex,^2^, ^5^, ^7^ leading to approval by the US Food and Drug Administration.

Cannabinoids, terpenes, flavonoids, and other compounds vary by strain and may work independently, antagonistically, or synergistically to produce different beneficial and adverse effects.^8^, ^9^ One recent preclinical study supported synergistic anti‐seizure efficacy of CBD and THC in a kindling model.^9^ We assessed the tolerability and efficacy of low‐THC/high‐CBD (1 T:20C and/or 1 T:50C) formulations to treat convulsive seizures for children and adults with diverse TREs.

## Methods

This prospective observational study was conducted at The Center for Discovery (TCFD), a residentially based program located in upstate New York. IRB approval was obtained to collect and analyze data on patients who were certified for medical cannabis (MC). For patients who lacked intellectual capacity, legal guardians consented. Per the New York State (NYS) Medical Marijuana Program, a registered physician completed the NYS Department of Health (NYSDOH) Medical Marijuana course and registered with the NYSDOH Medical Marijuana Program. Per DOH guidelines, enrolled patients were then certified by the registered physician to receive a NYS‐approved MC formulation (Columbia Care, LLC). After certification, patients were registered with NYS and designated TCFD as a facility caregiver. The program has a high ratio of clinical staff to patients and routinely collects detailed data on all residents on neurological, medical, and behavioral/lifestyle factors.

Twenty‐nine patients were observed over > = 24 weeks (maximal 9 months) after a 90‐day pre‐intervention baseline phase. Inclusion criteria included enrollment in the residential program, treatment‐resistant childhood‐onset epilepsy, and all of the following: (a) TRE failure to control seizures despite an appropriate trial of two or more anti‐seizure medications (ASMs) at therapeutic doses; (b) video‐EEG characterization of current seizures; and, (c) during pre‐baseline, > = 1 convulsive (atonic, tonic, tonic–clonic, or focal motor) seizure per month over a 90‐day consecutive period (e.g., absence and myoclonic seizures were not counted) (Table 1).

**Table 1:** Demographic and clinical features

Baseline characteristics	
Female (%)	12 (41.4%)
Male (%)	17 (58.6%)
Mean Age (Range)	25.6 (12.2–45.8)
Mean Age, Female (Range)	26.8 (12.2–42.0)
Mean Age, Male (Range)	24.8 (18.2–45.8)
Mean BMI at Baseline (Range)	22.09 (16.5–34.2)
Mean BMI at Baseline, Female (Range)	3 (10.3.28 (12.5–26.4)
Mean BMI at Baseline, Male (Range)	21.82 (16.5–34.2)
Lennox–Gestaut Syndrome	11 (37.9%)
Focal or Multifocal Epilepsy	15 (51.7%)
Generalized Seizures	3 (10.3%)
Number of Unique Current ASMs	20
Anti‐seizure Therapies at Baseline – *n* (%)	
Clobazam	12 (41.4%)
Brivaracetam	4 (13.8%)
Carbamazapine	3 (10.3%)
Clonazepam	6 (20.7%)
Diazapam	7 (24.1%)
Eslicarbazepine	1 (3.4%)
Felbamate	3 (10.3%)
Gabapentin	1 (3.4%)
Lacosamide	6 (20.7%)
Lamotrigine	12 (41.4%)
Levetiracetam	11 (37.9%)
Lorazepam	3 (10.3%)
Mysoline	1 (3.4%)
Oxcarbazepine	1 (3.4%)
Perampanel	3 (10.3%)
Phenobarbital	3 (10.3%)
Rufinamide	5 (17.2%)
Topiramate	3 (10.3%)
Valproate	6 (20.7%)
Zonisamide	3 (10.3%)
Vagus nerve stimulation	1 (3.4%)

### Patient history

Patients failed an average of 10 ASMs. All 29 patients had convulsive seizures. Five patients were treated with a vagus nerve stimulator (VNS): one active and four explanted or inactive. Six patients used the ketogenic diet, and four patients had neurosurgery.

Psychiatric medications, if used, were stable for 4 weeks prior to enrollment. The one patient with an active VNS had stable settings for a > = 90 days. Exclusion criteria included any of the following: (a) epilepsies associated with progressive or neurodegenerative diseases (e.g., neuronal ceroid lipofuscinosis, progressive myoclonus epilepsies, Rasmussen encephalitis, and tumors); (b) epilepsies associated with an inborn error of metabolism (e.g., mitochondrial disorders); and, (c) felbamate initiated within the past 6 months. Others were excluded if they had extended hospital stays with interruption of MC therapy.

The MC certified for all patients was ClaraCeed, a low‐THC/high‐CBD (1 T:20C) and/or ClaraCeed Ultra, (1 T:50C) tablet that, as per protocol, included a maximal dose of 6 mg THC/per day regardless of ratio. Ingredients included: Microcrystalline Cellulose, Dicalcium Phosphate, Silicon Dioxide, Magnesium Stearate, Talc, Sodium Starch Glycolate, Fractionated Coconut Oil MCT. All patients in this analysis were started on 2 mg/day of THC at a low‐THC/high‐CBD preparation of MC ratio of 1 T:20C ratio. In all cases, patients included in the study analysis were increased by 2 mg/day of THC every 7 days up to a maximum dose of 6 mg/day of THC of the 1:20 tablet. Patients were on 3 mg of THC and 60 mg of CBD twice daily on study day 15. They were then observed by their clinical team on the dose determined to provide the best balance of efficacy and tolerability.

**Figure 1:**
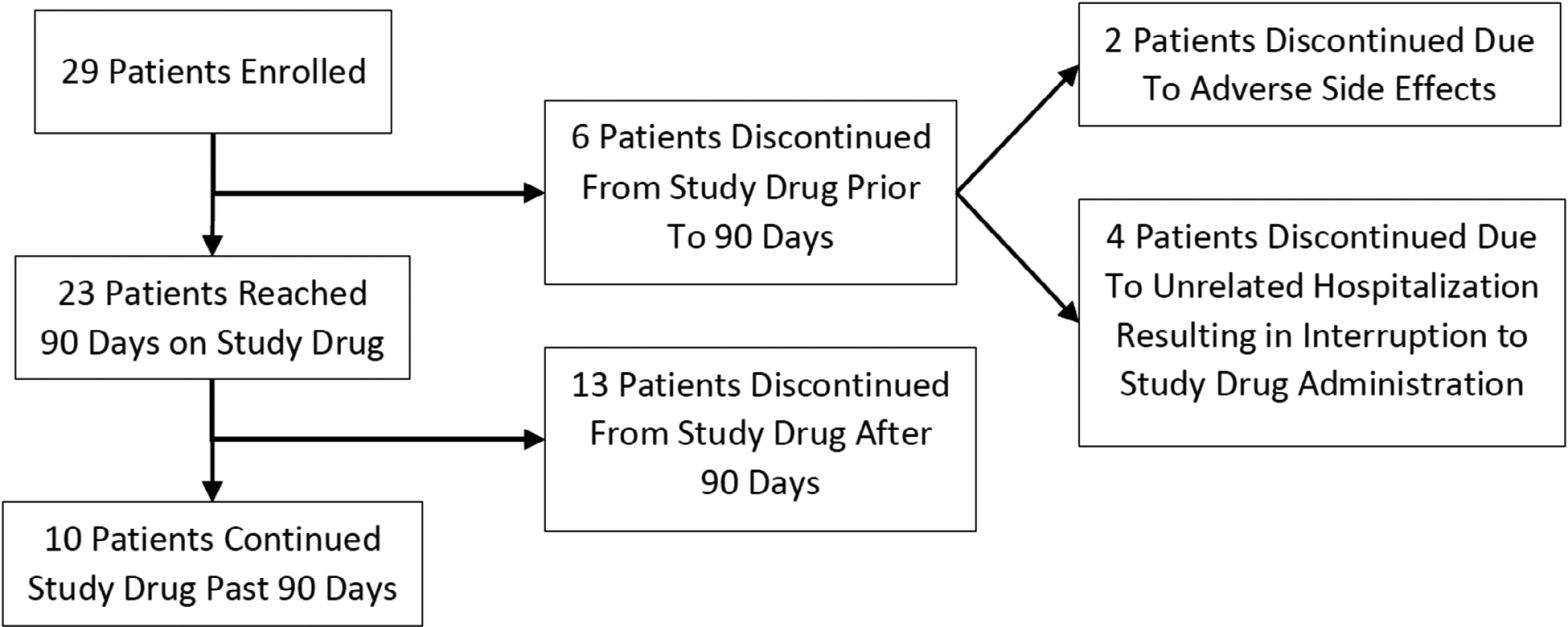
Study flow chart.

If the efficacy after full titration of the 1 T:20C was incomplete by week 24, but study medication was well tolerated, patients were moved to a higher CBD:THC ratio, 1 T:50C, and titrated to a maximal dose of 6 mg/day of THC of the 1 T:50C (i.e. 6 mg/day of THC and 300 mg/day of CBD), as tolerated. Safety and tolerability evaluations were conducted at baseline, and every week for the first month or until maximal tolerated dose was achieved, then once a month through the end of the study period. Caregivers and clinical staff routinely document seizure frequency, duration, use of rescue medications, and recovery duration. All data were entered into the patient's record. Patients were also assessed for secondary factors including concomitant ASM levels a minimum of three times during the study period at weeks 1, 12, and 24. The concomitant ASMs remained stable for the first 4 weeks before study onset, except for Felbamate, which was required to be stable for 6 months before initiation. Figure 1 is a flow diagram of the study subjects.

### Data records

Twenty‐nine patients were observed for a 90‐day pre‐intervention review for seizure frequency of at least one countable (i.e. convulsive) seizure per month. Pre‐intervention data included 90 days of seizure history. Mean monthly convulsive seizure frequency was 16.1 (range, 1–106). Pre‐intervention baseline data for all subjects included primary and secondary outcome measures over 90 days before first dose of study medication included: (1) seizure type, (2) seizure frequency, (3) seizure duration, (4) rescue medication use, (5) postictal duration, (6) nightly sleep data, (7) bowel movement data, (8) maladaptive behavior data for patients who with behavioral problems and a behavioral intervention plan, (9) blood pressure, pulse and weight, (10) neurological examination, and (11) complete blood counts and comprehensive metabolic tests.

During the study period, seizure type, date, time, duration, intervention use (e.g., PRN/Rescue Medication, ASMs, etc.), and postictal duration were documented for each seizure. TCFD routinely collects data on sleep and bowel elimination for each 24‐hour period. All anti‐seizure medications administered including PRNs were recorded in the medical record. For patients with preexisting behavioral intervention plans, daily behavioral data were recorded; behavioral data were not recorded for those without a behavioral intervention plan. Vital signs, physical, and neurological examinations were conducted at each physician visit. In‐person physician visits were held once weekly from the first administration of MC through the achievement of the maximal dose, and bi‐weekly thereafter through a combination of in‐person and telephonic visits.

## Statistical Analysis

### Software

All analyses were completed using the R programming language version 4.0^10^ and the RStudio integrated development environment.^11^ Data cleaning was performed using the dplyr^12^ and tidyr^13^ packages. Graphics were created using the ggplot2 package.^14^

Normality was assessed using histograms and measures of spread (i.e., skew and kurtosis). Data were not normally distributed and required nonparametric analyses. Seizure data were analyzed using a Wilcoxon rank sum test due to the small number and non‐normal distribution.

### Phases

This study included three phases: (1) pre‐treatment; (2) titration; and, (3) stabilization on the maximal dose predetermined using a 1 T:20C formula. The pre‐treatment phase included 90‐days before the first dose of the study medication. The titration phase comprised the period from the first administration of the study medication until the maximal tolerated dose was determined. The stabilization on maximal dose phase included the first 90‐days following the maximal dose, unless the patient was removed from treatment before this time. The maximal dose was the most stable dose utilized during the study period on a 1 T:20C formula, that is, the dose of CBD and THC identified as most effective and best tolerated during the titration period (Fig. 1).

We compared the pre‐treatment phase and the stable maximal dose phase. The titration phase was excluded due to the variable dosing required for each patient.

### Outcomes

The primary outcome was change in convulsive seizure frequency from the pre‐treatment baseline to the stable maximal dose phase. Secondary outcomes were seizure duration and seizure recovery time, which were recorded in minutes. Use of rescue medications, behavior data, sleep, and bowel elimination routinely kept records were analyzed as additional measures of treatment efficacy.

### Results

The 29 patients ranged in age from 12.2 to 45.8 years (mean, 25.6 years); 17 were female. Patients' epilepsy diagnoses included: 11 ‐ Lennox–Gastaut; 15 ‐ focal or multifocal; and three ‐generalized.

The average dose per subject on the maximal dose phase was 5.9 mg THC and 117.2 mg CBD. The average dose per subject in mg/kg per day was 0.11 mg/kg/day for THC and 2.3 mg/kg/day CBD.

**Figure 2:**
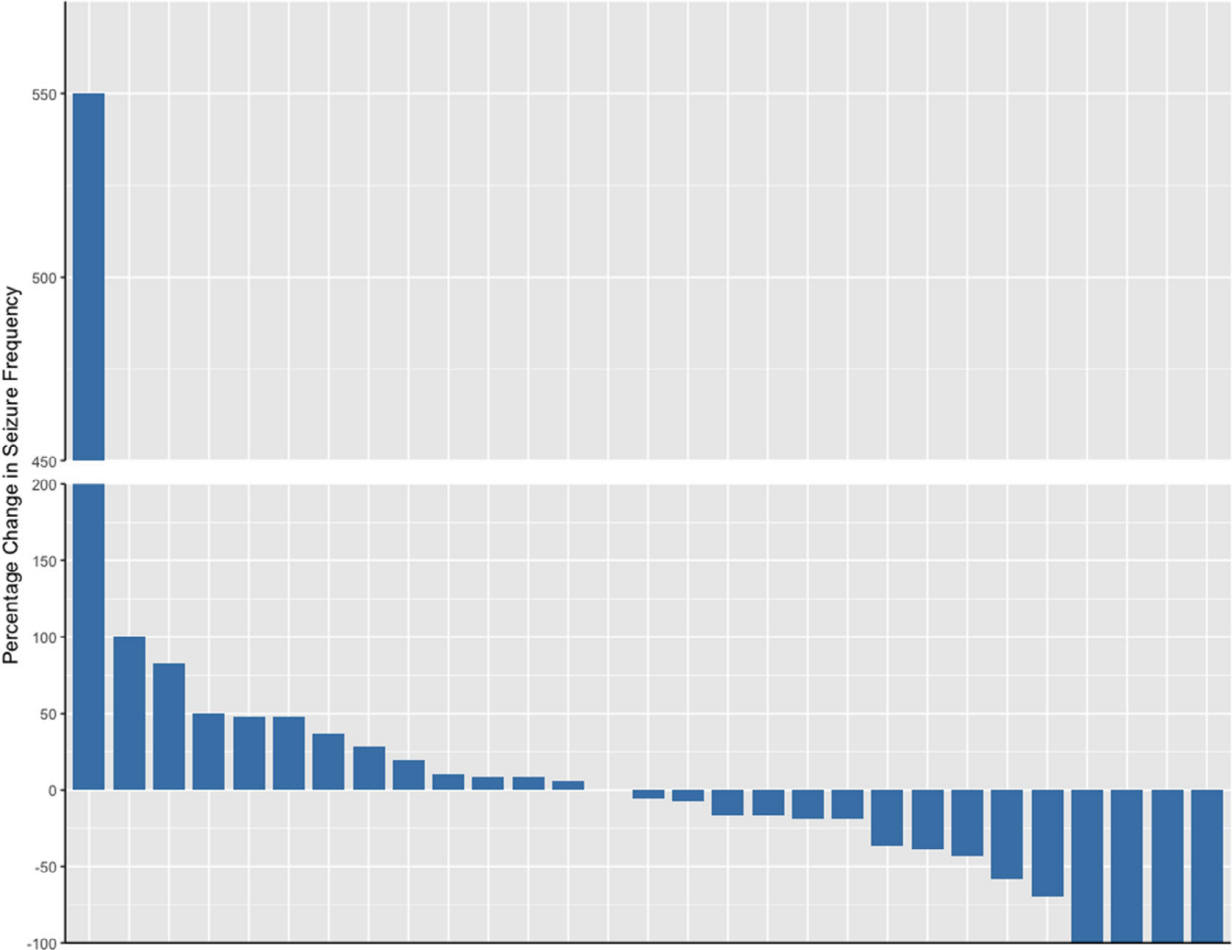
Percentage change in convulsive seizure frequency, *n* = 29. Bar represents changes in seizure frequency from pre‐intervention to maximal dose periods for each patient. The patient without change in seizure frequency is in the space without a bar.

**Figure 3:**
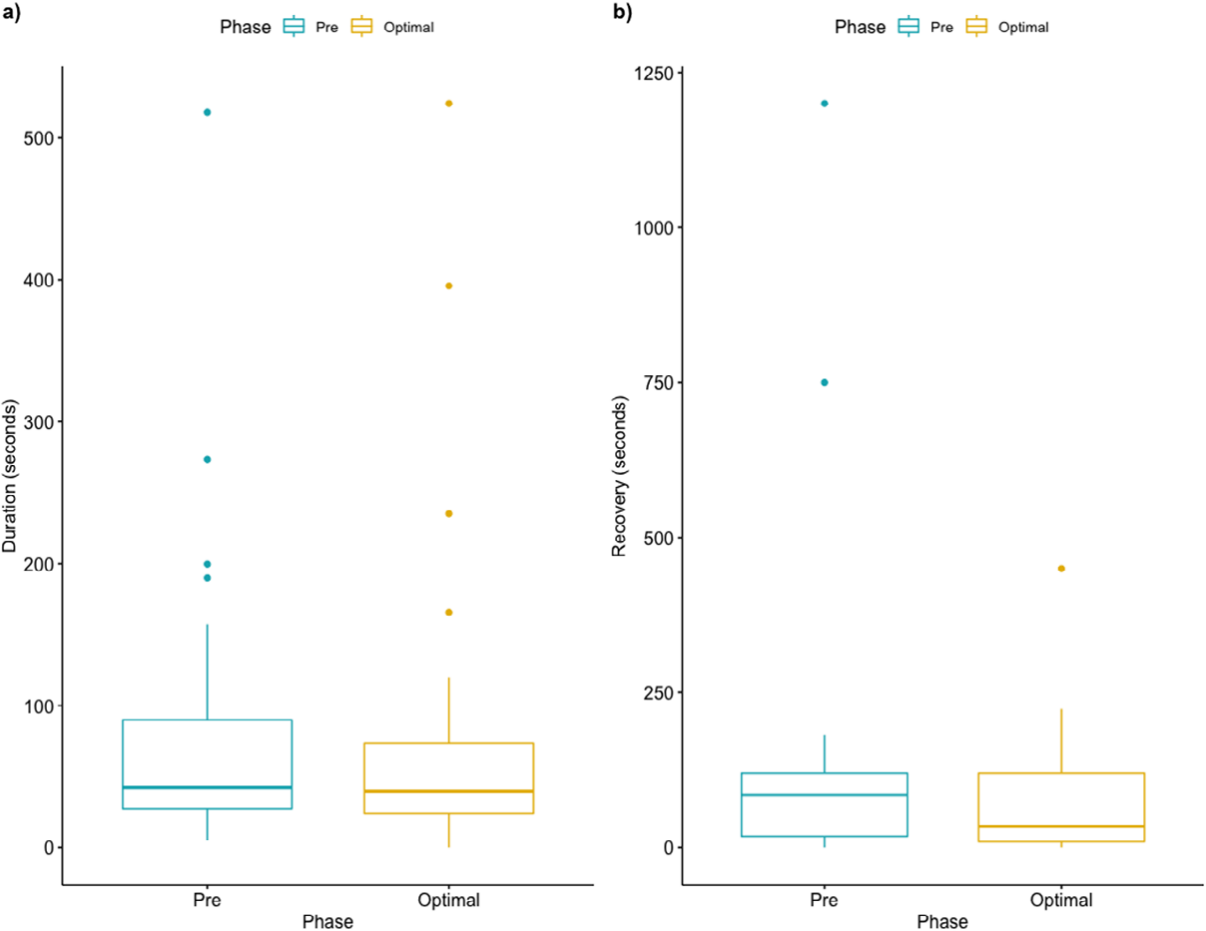
(A) Change in average duration of seizures between baseline and optimal dose periods. (B) Change in average duration of postictal state between pre‐intervention and optimal dose period.

Primary Outcome: Figure 2 summarizes the percent change in convulsive seizure frequency and Figure 3 summarizes the percent change in the duration of seizures and postictal periods. The median change from baseline to maximal dose period was −5.3% (IQR = − 39% – 28.6%; there was no statistically significant difference average seizure frequency between the maximal and pre‐intervention phases using a Wilcoxon rank sum test with p = 0.99. Six patients (21%) had a ≥ 50% reduction in convulsive seizures. Of that six, four patients (13.8%) became free of convulsive seizures during the maximal dose period. Four patients (13.8%) had a ≥ 50% increase in convulsive seizures during the maximal dose period.

Median change in seizure frequency between baseline and maximal dose period −3 recorded seizures, change in IQR −11. There was no statistically significant difference found in frequency of seizures between the optimal and pre‐intervention phases using a Wilcoxon rank sum test (Fig. 2).

*A*. Median changes in average seizure duration between baseline and maximal dose periods was –2.5 seconds (IQR −12.8 seconds; *p* = 0.44). B. *C*hange in average duration of postictal state between pre‐intervention and maximal dose periods. Median change in postictal state duration was −50.7 seconds (QR – 9 secs; *p* = 0.26).

Excluding the four seizure‐free patients, we found no differences in the average duration of seizures (Fig. 3A) or postictal period (Fig. 3B).

**Figure 4:**
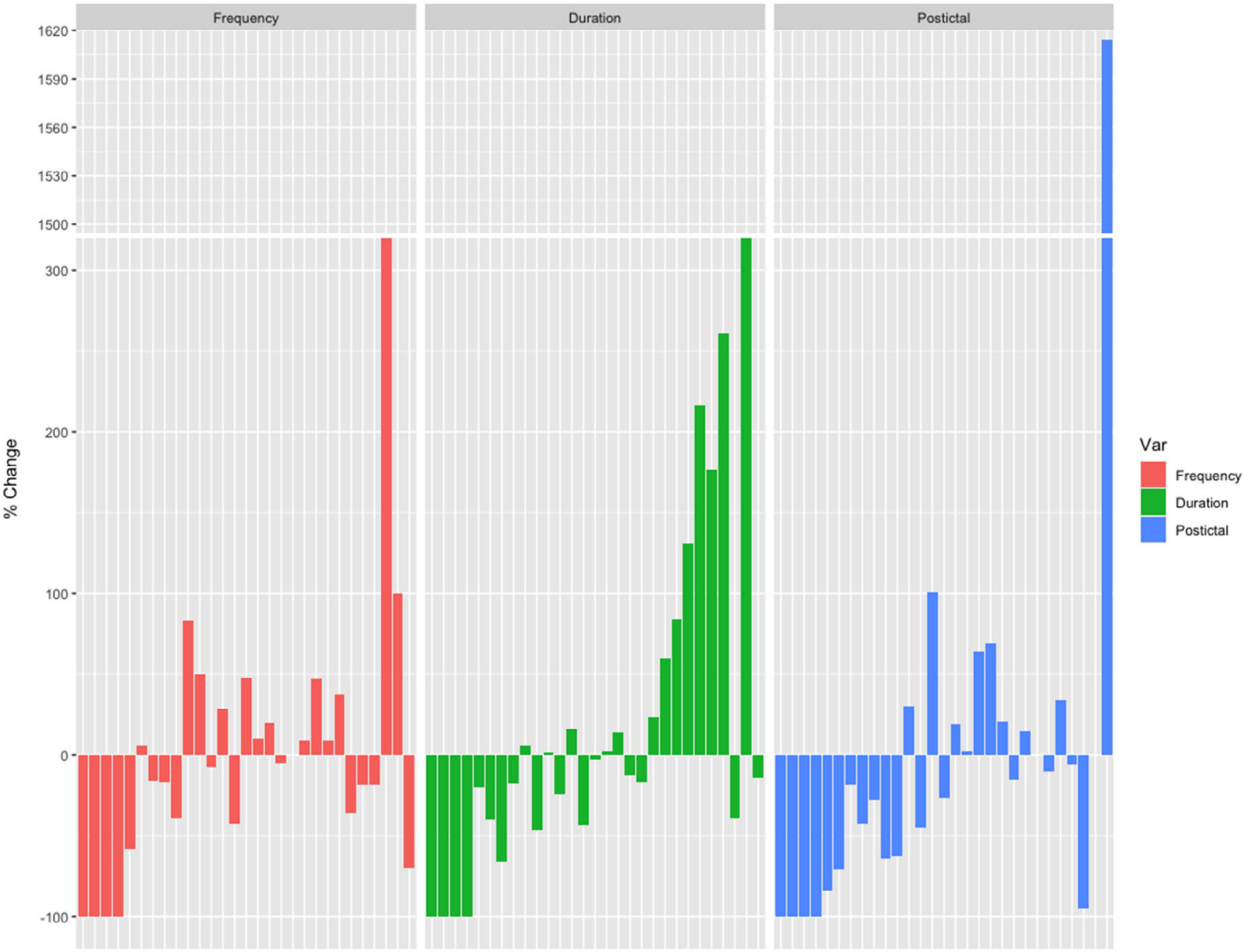
Percentage change in seizure frequency, mean seizure duration, and mean postictal duration between.

Figure 4 shows the changes in seizure frequency, seizure duration, and postictal duration. Seven patients had a decrease in all three metrics; 11 patients decreased in two metrics; five decreased in one metric. Six patients had an increase in seizure frequency, duration, and postictal state; four increased across two metrics; 12 increased in one metric.

**Figure 5:**
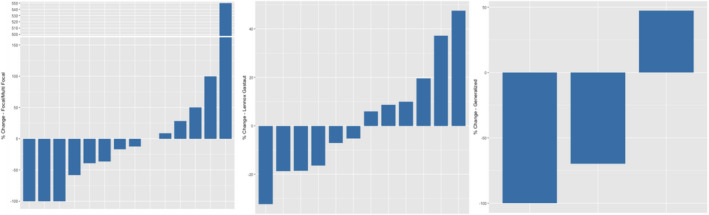
Changes in seizure frequency by epilepsy syndrome.

Figure 5 shows the changes in seizure frequency for individual patients with Focal/Multifocal Epilepsies (left), Lennox–Gastaut Syndrome (middle), and Generalized Epilepsies (right). There were no significant difference in reduction of seizure frequency or duration or postictal duration when the three different epilepsy syndromes were analyzed.

### Rescue medication

We found no difference in rescue medication use between baseline and maximal dose periods (*p* = 0.66). Median change in percentage of required rescue medication between the baseline and maximal dose period was −10% (IQR ‐100%–300%).

### Potential influence of clobazam

Since CBD is a potent inhibitor of cytochrome 2C19 and can thereby elevate levels of the active clobazam metabolite, desmethyl‐clobazam, we assessed the 12 patients on clobazam. There was no signal of improved efficacy in patients on clobazam for seizure frequency or duration or postictal duration.

### Efficacy by syndrome

We assessed patients by the major epilepsy syndromes in our cohort (Focal or Multifocal, Lennox–Gastaut Syndrome, Generalized Epilepsies) (Figure 4). There were no significant differences in these groups, although there was a trend for reduced postictal state in the Focal/Multifocal and Generalized Epilepsy groups, as nine individuals had a > =50% decrease and one had a > =50% increase in postictal state.

### Anti‐seizure medications

Thirteen anti‐seizure medication doses were reduced in seven patients, including benzodiazepines in six patients. Other reduced ASMs included phenobarbital, felbamate, levetiracetam, perampanel, lamotrigine, and zonisamide. For one patient, reductions occurred during the observation period and six afterwards. There were no increases of ASM during the observational period.

### Adverse events

Ten adverse events were reported in 6/29 (20.7%) patients, including emesis (n = 2 [6.9%]), gums/nose bleeding (n = 2 [6.9%]), upper respiratory infection (n = 1 [3.4%]), minor upper respiratory distress (n = 1 [3.4%]), fatigue (n = 1 [3.4%]), dizziness (n = 1 [3.4%]), and status epilepticus (n = 1 [3.4%]). Two adverse events caused Emergency Department evaluations, including respiratory distress and status epilepticus. There were 16 serious adverse events, all deemed unrelated to the study medication, in 7/29 (24.1%) patients. Serious adverse events included pneumonia (n = 5 [17.2%]) and ileus (n = 1 [3.4%]) and required hospitalization in two patients. Two patients were removed from the study due to serious adverse events: increased seizure frequency and increased maladaptive behaviors.

No adverse or serious adverse events were deemed related to the study drug, and were consistent with patient's histories and diagnoses.

### Additional metrics

Twelve patients had behavioral disorders before study onset requiring behavioral tracking plans. Median percentage change in target behavioral episodes was −7.3% (IQR –16.1%–6.5%); there was no difference in average behavioral episodes between the maximal dose and pre‐intervention phases (*p* = 0.2). Nine had reduced target behaviors and three had increased target behaviors during maximal dose v. baseline periods.

There was no significant change in sleep duration between baseline and maximal dose periods using a Wilcoxon rank sum test with *p* = 0.33; median percentage change was −2.6% (IQR, −5% to 5%). No patient had a ≥50% change in average duration of sleep.

There was a marginally significant difference in frequency of bowel movements between the maximal dose and pre‐intervention phases using a Wilcoxon rank sum test with *p* = 0.064 a trend of more frequent bowel movements.

There were no consistent or clinically significant abnormalities in blood cell counts or metabolic tests, including liver function studies, in our patients during the study period.

## Discussion

This prospective observational study of a low‐THC/high‐CBD (1 T:20C or 1 T:50C) formulation in diverse TREs revealed that these MC formulations were well tolerated up to a predetermined maximum dose, but did not significantly reduce seizure frequency or duration, or postictal duration when analyzed at the group level. Similarly, at the group level comparing baseline and treatment periods, we found no significant differences in rescue medication use, maladaptive behavioral episodes, or sleep duration, although there was a trend for more frequent bowel movements during treatment. All patients achieved their predetermined maximal doses using a 1 T:20C formula: 27 patients achieved a 6‐mg THC with 120 mg CBD daily dose; two patients achieved a 4‐mg THC with 80 mg CBD daily dose. Although some patients achieved this dose of 1 T:50C beyond the 90‐day analysis period. For most patients on the 1 T:20C formulation, the daily doses of THC were higher than most previous studies, but the dose of CBD was lower, averaging 2.3 mg/kg/day of CBD. By contrast, Epidiolex is approved for Dravet and Lennox–Gastaut Syndromes with target doses of CBD at 10–20 mg/kg/day.

There was considerable variability in convulsive seizure frequency, with some patients experiencing >50% increases or decreases during the treatment period. Similarly, seizure duration and postictal durations varied markedly between baseline and treatment periods. We cannot distinguish spontaneous variability from positive or negative effects of MC among individual patients. It is possible that both natural fluctuations as well as heterogeneous effects of MC contributed to the observed variation. Among those with increased seizure frequency or duration, or postictal dysfunction, all returned to baseline in these metrics after MC was reduced or eliminated, therefore, there were no lasting negative impacts from the trial. This observation can support either regression to the mean or reversible adverse effects of MC, or both.

Of the 29 patients in the study, seven had their ASMs doses reduced during MC therapy, however, only one experience had ASMs decreased during the observational period while six had decreased after the observational period. No patients required increased ASMs while receiving the MC. The potential benefits of decreased ASMs in this population of highly medically complex patients deserves further study.

Although there was no significant difference between rescue medication use between pre‐intervention and maximal dose phases at the group level, a third of patients had a ≥50% reduction in use of rescue medications with five of these 18 requiring no rescue medication during the optimal phase. Conversely, four patients who received no rescue medications during baseline required them during the optimal phase, although all had received rescue medications in past. We found no significant difference in sleep duration between the pre‐intervention and optimal phases. There was a trend for increased frequency of bowel movements during MC treatment. Since diarrhea is a potential adverse effect of higher dose CBD^5^ this could reflect a milder effect, which was beneficial in those with constipation.

Adverse events among the patients on MC during the optimal dose phase was similar to baseline and patient's prior records. Overall, the study drug was well tolerated by most patients. One patient on 6 mg THC and 120 mg CBD daily had increased maladaptive behaviors necessitating removal from the study during the 90‐day observation period. The behaviors returned to baseline after the medication was stopped. These behaviors may result from THC since the CBD dose was relatively low and was rarely associated with adverse behavioral changes even at much higher doses. Another patient had increased seizure frequency on MC leading to discontinuation. Seizure frequency for this individual returned to baseline after MC was stopped. Unrelated to study drug toleration, four patients were removed before the completing the observation period because of unrelated hospitalizations. In summary, six patients were removed before completing the 90 days on the maximal dose.

This study was limited by lack of blinding and small sample size. However, the study benefited from unusually structured and near‐continuous observation at TCFD before and during the study period, with detailed assessments of seizure activity as well as behavior, sleep, and other measures. Although this study was not powered to identify small but statistically significant reductions in seizure frequency or other measures, we found no trend for reduced seizure frequency or duration, suggesting that a larger sample may not yield a statistically significant difference. Two randomized controlled trials of CBD and the closely related CBDV for focal epilepsy reported was equivalent to placebo.^15^ Our study was constrained by the initial availability of only the 1 T:20C dosing formulation, which resulted in a relatively high THC and low CBD dose compared to prior studies. The doses of CBD used in our trial were lower than those that showed efficacy on earlier open‐label and randomized controlled trials in Dravet, Lennox–Gastaut, and Tuberous Sclerosis Complex Syndromes; 2.3 mg/kg/day versus 10‐ > 20 mg/kg/day.^3^, ^4^, ^5^, ^6^, ^7^, ^16^ However, the doses used in our study are consistent with doses commonly employed by patients at cannabis dispensaries. For example, in New York State, a 1‐month supply of CBD at our dosing would cost $385–800 dollars, depending on the manufacturer and also the THC content.

Our data suggest that THC at doses of up to 6 mg daily did not reduce seizure frequency or severity in our patients. There are an infinite number of ratios and targeted doses for THC and CBD, but it may be worthwhile assessing higher ratios of CBD:THC (e.g., 1:50) in populations with well‐defined epilepsy syndromes. The trend for reduced postictal duration deserves further study. Few investigations assess postictal duration, which may be a marker of seizure severity and long‐term cumulative effects of recurrent seizures on brain function.

Overall, the MC formulations were well tolerated but had no significant reductions in seizure frequency, duration, postictal time, or recue medication use at the group level. Ten individuals continued on a‐1 T:50C formulation, with six experiencing a decrease in their ASMs after the study period, which supports potential benefits for a subset of our population. Given the widespread use of MC formulations to treat epilepsy patients with both CBD and THC in state dispensaries, our findings suggest that larger, randomized controlled studies should be conducted to more fully assess their safety and efficacy. Further, our study, one of the first to assess postictal state duration, rescue medication use, and behavioral changes should be replicated as these factors can significantly affect quality of life and be determining factors for use of MC or other therapies in TRE patients.

### Limitations

This study was limited by lack of blinding, heterogeneous patient population and epilepsy syndromes, small sample size, and predetermined maximal dose ratios of THC:CBD.

## Author Contributions

Persons responsible for the planning of the study, data collection and analysis, and manuscript preparation include Orrin Devinsky, MD; Theresa Hamlin, Ed.D; Philip Wilken, MD, MS; Angelica Marmanillo, BSN, RN; Daniel Ryan, M.Ed; and, Conor Anderson B.Eng. George J. Todd, MD, FACS served as the oversight medical advisor for TCFD. Daniel Friedman, MD provided supplemental analytics support and edited the manual.

## Conflict of Interest

This study was funded in part by Columbia Care, Inc. who produced the study drug used in this observational study. They were not involved in any aspect of the treatment protocols, data collection, or interpretation of results. They were selected by a team of physicians and clinicians based on the consistency and efficacy of their product over time. None of the investigators received an honorarium from Columbia Care.

## Supporting information


**Table S1** Percentage Change in Rescue Medication: Total and percentage change in rescue medication administered between pre‐intervention and optimal dose period, n = 18.

**Table S2** ASM Reduction in Dosage: Of the 29 patients in the study, seven patients decreased their dosages of anti‐seizure medications while receiving MC: one decreased during the 6 months allotted for the analysis, and six decreased after.
