# Supplementary material for: Observational study of medical marijuana as a treatment for treatment‐resistant epilepsies
Source: Ann Clin Transl Neurol. 2022 Mar 10;9(4):497–505. doi: 10.1002/acn3.51537 (PMC8994986; doi:10.1002/acn3.51537)
Supplement: Supplementary file 1 — Table S1 Percentage Change in Rescue Medication: Total and percentage change in rescue medication administered between pre‐intervention and optimal dose period, n = 18. [file ACN3-9-497-s002.pdf]

Table S1

Patient	Total Pre-Intervention	Total Optimal Dose	Total Change	% Change
1	1	0	-1	-100
2	13	5	-8	-61.54
3	5	4	-1	-20
4	5	7	2	40
5	0	2	2	Inf
7	15	16	1	6.67
8	1	3	2	200
10	0	1	1	Inf
11	0	5	5	Inf
12	1	0	-1	-100
13	1	4	3	300
16	0	2	2	Inf
18	4	3	-1	-25
20	6	4	-2	-33.33
22	1	0	-1	-100
25	1	0	-1	-100
28	2	0	-2	-100
30	1	1	0	0
