# Supplementary material for: Observational study of medical marijuana as a treatment for treatment‐resistant epilepsies
Source: Ann Clin Transl Neurol. 2022 Mar 10;9(4):497–505. doi: 10.1002/acn3.51537 (PMC8994986; doi:10.1002/acn3.51537)
Supplement: Supplementary file 2 — Table S2 ASM Reduction in Dosage: Of the 29 patients in the study, seven patients decreased their dosages of anti‐seizure medications while receiving MC: one decreased during the 6 months allotted for the analysis, and six decreased after. [file ACN3-9-497-s001.pdf]

Table S2

Patient	MC Dose	ASM Medication	Pre-MC Dose	Reduced to
8	6mg, 1:50 ratio	Onfi	30mg/day	25mg/day
8	6mg, 1:50 ratio	Phenobarbital	162mg/day	146mg/day
8	6mg, 1:50 ratio	Felbamate	400mg/day	200mg/day
10	6mg, 1:50 ratio	Depakote	1750mg/day	1500mg/day
10	6mg, 1:50 ratio	Onfi	20mg/day	17.5mg/day
11	6mg, 1:50 ratio	Lamictal	1000mg/day	800mg/day
4	6mg, 1:50 ratio	Fycompa	10mg/day	8mg/day
4	6mg, 1:50 ratio	Onfi	60mg/day	30mg/day
4	6mg, 1:50 ratio	Zonegran	100mg/day	0 (discont.)
13	6mg, 1:50 ratio	Keppra	2000mg/day	1000mg/day
17	6mg, 1:50 ratio	Onfi	15mg/day	10mg/day
17	6mg, 1:50 ratio	Clonazepam	1mg/day	0.75mg/day
21	6mg, 1:50 ratio	Clonazepam	3mg/day	1mg/day
